# Integrative analysis of the therapeutic mechanisms of Astragaloside IV in idiopathic pulmonary fibrosis via network pharmacology and molecular validation

**DOI:** 10.1038/s41598-025-23354-8

**Published:** 2025-11-12

**Authors:** Shanshan Chen, Jin Yan, Jing Zhang, Yiwen Ma, Yiliang Su, Yiming Yu

**Affiliations:** Department of Respiratory and Critical Care Medicine, Tongren Hospital, Shanghai Jiao Tong University School of Medicine, No. 1111 Xianxia Road, Shanghai, 200336 China

**Keywords:** Astragaloside IV (AS-IV), Idiopathic pulmonary fibrosis, PI3K-AKT signaling pathway, PIK3CA, Drug discovery, Molecular biology, Diseases

## Abstract

Astragaloside IV (AS-IV), an essential active ingredient isolated from Astragalus membranaceus, has exhibited notable antifibrotic properties in diverse disease models. However, its exact role and underlying molecular mechanisms in idiopathic pulmonary fibrosis (IPF) remain insufficiently defined. In this study, we employed an integrative approach combining transcriptomic profiling, network pharmacology, and molecular docking techniques to investigate the critical targets and signaling pathways modulated by AS-IV in the context of IPF. Differentially expressed genes obtained from lung tissue datasets of IPF patients were cross-referenced with AS-IV-associated targets to identify overlapping candidates with potential therapeutic relevance. Gene enrichment analyses revealed that the PI3K-AKT pathway plays a central role in mediating AS-IV’s biological effects. Molecular docking and subsequent dynamic simulations demonstrated that AS-IV binds stably to the PIK3CA protein. In vitro experiments confirmed that AS-IV suppressed the activation of fibroblasts and decreased the expression of fibrotic markers. Furthermore, in a bleomycin-induced mouse model, AS-IV administration significantly reduced collagen accumulation and pulmonary fibrosis. These results collectively suggest that AS-IV exerts protective effects against IPF progression primarily through modulation of the PI3K-AKT pathway by targeting PIK3CA, supporting its potential development as a novel antifibrotic therapeutic agent.

**Supplementary Information:**

The online version contains supplementary material available at 10.1038/s41598-025-23354-8.

## Introduction

Idiopathic pulmonary fibrosis, a long-term and worsening interstitial lung disease, typified by persistent alveolar inflammation and extensive fibrotic changes that progressively impair lung function and exacerbate breathing difficulties. As the most prevalent form of pulmonary fibrosis, untreated IPF has a median survival of merely 3–5 years^1^. Present therapeutic strategies are primarily focused on decelerating disease advancement, alleviating symptoms, and improving patients’ quality of life. Although antifibrotic agents such as Nintedanib and Pirfenidone have demonstrated the ability to slow the decline in lung function, their clinical utility is constrained by high costs and potential side effects^2,3^. In cases of advanced disease, lung transplantation may be the sole curative option; however, it remains applicable only to a limited group of patients due to strict eligibility and donor availability^4^. Thus, there is a pressing demand for innovative, effective, and widely accessible treatment solutions.

An increasing body of clinical and preclinical evidence supports the therapeutic value of traditional Chinese medicine (TCM) in mitigating pulmonary fibrosis, offering a promising adjunctive approach to managing this complex disease^5^. Among various TCM constituents, Astragaloside IV (AS-IV)—a principal saponin derived from *Astragalus membranaceus*, a classical Chinese medicinal herb—has attracted substantial interest. AS-IV is chemically categorized as a triterpenoid glycoside, consisting of an aglycone moiety conjugated to glucose and rhamnose. It exhibits multiple pharmacodynamic actions, including anti-inflammatory, antioxidative, and immune-modulating effects^6^. Despite these promising attributes, the precise mechanisms by which AS-IV alleviates pulmonary fibrosis remain to be fully elucidated.

Traditional pharmacological approaches often emphasize a “one drug–one target–one disease” paradigm. In contrast, network pharmacology offers a holistic and integrative methodology capable of capturing complex drug–target–pathway interactions. This systems-level framework is well-aligned with the foundational principles of TCM, which emphasize multi-component synergy and syndrome differentiation^7^. Network pharmacology is especially suitable for studying multifactorial diseases such as IPF. Building upon this concept, the present study aims to systematically identify the molecular targets and signaling pathways modulated by AS-IV in IPF, integrating transcriptomic profiling, network-based prediction, and experimental validation to provide mechanistic insights and potential clinical translation strategies.

## Materials and methods

### Network pharmacology analysis

#### Selection of differential genes in IPF

The GEO database (https://www.ncbi.nlm.nih.gov/geo) was searched using the keyword “Idiopathic Pulmonary Fibrosis, IPF” to retrieve gene expression profiles. The dataset GSE53845 was selected, which includes 40 IPF lung tissue samples and 8 healthy controls^8^. Following the acquisition of the normalized expression matrix, differential gene expression was analyzed via the ‘limma’ package in R, employing criteria of |log2FC|> 0.4 and *p*-value < 0.05 for gene selection.

#### Collect IPF targets

IPF-related targets were collected from multiple databases, including GeneCards, OMIM, PharmGKB, TTD, DisGeNET, and DrugBank, using “Idiopathic Pulmonary Fibrosis” as the search term. After removing duplicates, the combined set of genes was considered disease-related targets. A Venn diagram was generated to visualize intersections and unique targets among databases.

#### Identification of active constituents of AS-IV and prediction of potential targets

For AS-IV, active components were identified via the TCMSP database, filtered by pharmacokinetic parameters such as oral bioavailability (OB ≥ 20%) and drug-likeness (DL ≥ 0.18). Its molecular structure was obtained from the PubChem database. Target prediction was performed using SUPER-PRED and Swiss Target Prediction. All predicted targets were normalized to gene symbols for integration with disease data.

#### Target acquisition

Intersection analysis was conducted among the predicted AS-IV targets, IPF-related DEGs, and disease-associated genes. The overlapping genes were visualized via a Venn diagram and considered as potential therapeutic targets of AS-IV in IPF.

#### Functional enrichment analysis

Functional annotation, including Gene Ontology (GO) for biological processes, cellular components, and molecular functions, as well as Kyoto Encyclopedia of Genes and Genomes (KEGG) pathway enrichment, was performed with the ‘clusterProfiler’ package in R. Terms with *p*-values below 0.05 were deemed significant and utilized to infer the molecular mechanisms by which AS-IV may exert therapeutic effects against IPF.

#### Analysis of target gene interactions and key gene screening

Shared targets were uploaded to the STRING database (https://string-db.org/), specifying “Homo sapiens” as the species and applying a minimum confidence score of 0.4. The resulting protein–protein interaction (PPI) network was visualized using Cytoscape software. Key hub genes were identified using the CytoHubba plugin, based on parameters including degree, betweenness, and closeness centrality. Genes with high centrality values were regarded as core regulatory elements in IPF pathogenesis.

#### Molecular docking and molecular dynamics simulation

The crystal structure of PIK3CA (PDB ID: 8EXL) was downloaded from the Protein Data Bank^9^ and the 3D structure of AS-IV was retrieved from PubChem^10^ and optimized using the MMFF94 force field. Molecular docking was conducted using AutoDock Vina 1.1.2^11^. with the exhaustiveness parameter set to 32. The conformation exhibiting the lowest binding energy was selected as the starting point for molecular dynamics (MD) simulations.

The best-scoring protein–ligand complex was used as the starting structure for MD simulation using AMBER 18^12^. The system underwent 2500 steps of steepest descent and conjugate gradient minimization, then was heated to 298.15 K under NVT for 200 ps, followed by 500 ps of NVT and 500 ps of NPT equilibration. A 100 ns production run was performed under NPT conditions. Long-range electrostatic interactions were treated using the Particle Mesh Ewald (PME) method^13^, and hydrogen bonds were constrained using the SHAKE algorithm^14^, the Langevin thermostat was used for temperature control^15^, with the collision frequency γ set to 2 ps⁻^1^. The system pressure was 1 atm, the integration time step was 2 fs, and trajectories were saved every 10 ps for subsequent analysis.

#### MM/GBSA binding free energy calculation

Binding free energy between AS-IV and the protein was calculated using the MM/GBSA method^16,17^. Long molecular dynamics simulations may negatively impact the accuracy of MM/GBSA calculations; therefore, in this study, MD trajectories from 45–50 ns were used for the calculations. The specific formula is as follows:

$$\begin{aligned} \Delta G_{bind} = & \Delta G_{complex} - \left( {\Delta G_{receptor} + \Delta G_{ligand} } \right) \\ = & \Delta E_{{{\text{int}} ernal}} + \Delta E_{VDW} + \Delta E_{elec} + \Delta G_{GB} + \Delta G_{SA} \\ \end{aligned}$$

In the formula, ${\Delta E}_{{{\text{internal}}}}$ represents the internal energy, ${\Delta E}_{{{\text{VDW}}}}$ represents the van der Waals interactions, and ${\Delta E}_{{{\text{elec}}}}$ represents the electrostatic interactions. Internal energy includes bond energy (Ebond), angle energy (Eangle), and torsion energy (Etorsion). ${\Delta G}_{{{\text{GB}}}}$ and ${\Delta G}_{{{\text{GA}}}}$ together represent the solvation free energy, where GGB is the polar solvation free energy, and GSA is the nonpolar solvation free energy.

For ΔG_GB, the GB model developed by Nguyen et al. was used for calculation (igb = 2)^18^. The nonpolar solvation free energy (ΔGSA) was calculated based on the product of the surface tension (γ) and the solvent-accessible surface area (SA), with ΔGSA = 0.0072 × ΔSASA^19^. Entropy changes were neglected in this study due to the high computational cost and low accuracy^20^.

### Experimental validation

#### The culture and handling of cells

Human lung fibroblasts (MRC-5 cell line, Cat. No. ZQ0006, Shanghai Zhongqiaoxinzhou Biotech, China) were maintained in DMEM supplemented with 10% fetal bovine serum (FBS) at 37 °C in a humidified incubator with 5% CO₂. Upon reaching 70–80% confluence, the medium was switched to serum-free DMEM and cultured for an additional 24 h. Cells from passages 2–3 were used in subsequent experiments. Fibrotic modeling was induced by treating cells with 10 ng/mL TGF-β1 for 48 h^21^.

#### Cell viability assay

Cell viability was evaluated using the CCK-8 assay. HLFs (5 × 10^3^ cells/well) were seeded in 96-well plates, and after cell attachment, the medium was replaced with fresh DMEM containing varying concentrations of AS-IV (0.1, 1, 10, 50, 100, and 1000 μM). After a 48-h incubation period, 10 μL of CCK-8 reagent was added to each well, followed by a 1-h incubation at 37 °C. Absorbance was measured at 450 nm using a microplate reader (Thermo Fisher Scientific, Model 1510).

#### Cell grouping

①Control group: treated with DMEM only; ②TGF-β1 group: treated with 10 ng/mL TGF-β1; ③TGF-β1 + AS-IV group: treated with 10 ng/mL TGF-β1 and 50 μM AS-IV; ④TGF-β1 + si-NC group: transfected with siRNA negative control, then treated with 10 ng/mL TGF-β1; ⑤TGF-β1 + si-PIK3CA group: transfected with siRNA targeting PIK3CA, then treated with TGF-β1; ⑥TGF-β1 + si-PIK3CA + AS-IV group: transfected with si-PIK3CA and treated with both TGF-β1 and 50 μM AS-IV.

#### Western blot

Total protein was extracted from HLFs and mouse lung tissues using RIPA lysis buffer (Beyotime, Shanghai, China). Protein concentration was quantified using a BCA protein assay kit (Servicebio, China). Equal quantities of protein samples were loaded onto 4–12% SurePAGE gels (GenScript, USA) for SDS-PAGE and then transferred onto PVDF membranes. Membranes were blocked with 5% bovine serum albumin (BSA) or 5% non-fat milk at room temperature for 1 h, followed by overnight incubation at 4 °C with primary antibodies (dilution 1:1000). The primary antibodies used were as follows: GAPDH (1:1000, ab8245, Abcam); α-SMA (1:1000, ab7817, Abcam); Collagen I (1:1000, ab260043, Abcam); Fibronectin (1:1000, ab7817, Abcam); PIK3CA (1:1000, ab40776, Abcam); PI3K (1:1000, ab191606, Abcam); p-PI3K (1:1000, ab182651, Abcam); AKT (1:1000, ab179463, Abcam); p-AKT (1:1000, ab192623, Abcam); TGF-β1 (1:1000, ab215715, Abcam); and IL-11 (1:1000, 55169-1-AP, Proteintech, China). Following TBST washes, HRP-conjugated secondary antibodies (1:1000) were applied for 1 h at room temperature. Detection was performed with ECL reagents, and images were captured using a Tanon 5200 imaging system. Band intensities were quantified using ImageJ software. Membranes were stripped and reprobed when necessary. All experiments were independently repeated at least three times.

#### Animal grouping and intervention

Six-week-old male C57BL/6 mice were purchased from Jiangsu Jicao Biotechnology (License: SCXK(SU)2023-0042) and housed under SPF conditions at the Hongqiao International Medical Research Institute, Shanghai Tongren Hospital. Mice were euthanized by intraperitoneal injection of 2% pentobarbital sodium at a dose of 150 mg/kg, in accordance with institutional animal care guideline^22^. Mice were randomly divided into four groups (n = 6/group): Control, BLM, BLM + AS-IV low-dose (50 μM), and BLM + AS-IV high-dose (100 μM). Bleomycin (BLM; 2 mg/kg, 40 μL/mouse) was administered intratracheally on Day 0 under anesthesia. The control group received saline. On Day 21, AS-IV was dissolved in 0.5% CMC and administered orally every other day for the low-dose group and daily for the high-dose group. The control and BLM groups received an equivalent volume of CMC. All treatments lasted until Day 35. At the endpoint, mice were sacrificed, and lung tissues were collected. Samples were either fixed in 4% paraformaldehyde for histological examination or stored at − 80 °C for molecular analyses. All animal experiments were conducted in accordance with institutional and national guidelines for the care and use of laboratory animals, and reported in compliance with the ARRIVE guidelines. This study has been approved by the Ethics Committee of Tongren Hospital (Ethics Approval Number: 2024-164).

#### Histopathological analysis

Lung tissues collected on Day 35 were fixed in 4% paraformaldehyde for 24 h, followed by paraffin embedding and sectioning. Sections were stained with hematoxylin and eosin (H&E) and Masson’s trichrome. Slides were scanned using a Leica slide scanner and assessed for fibrosis severity using the scoring criteria established by Hubner et al.^23^.

#### Statistical analysis

All statistical analyses were performed using GraphPad Prism 10 Version 10.3.1. Graphs were prepared using Adobe Illustrator 2023. Data are presented as mean ± SEM. One-way ANOVA and unpaired t-tests were used to assess significance. A *p*-value < 0.05 was considered statistically significant.

## Results

### Identification of common targets between AS-IV and IPF

#### Differentially expressed genes

**Fig. 1:**
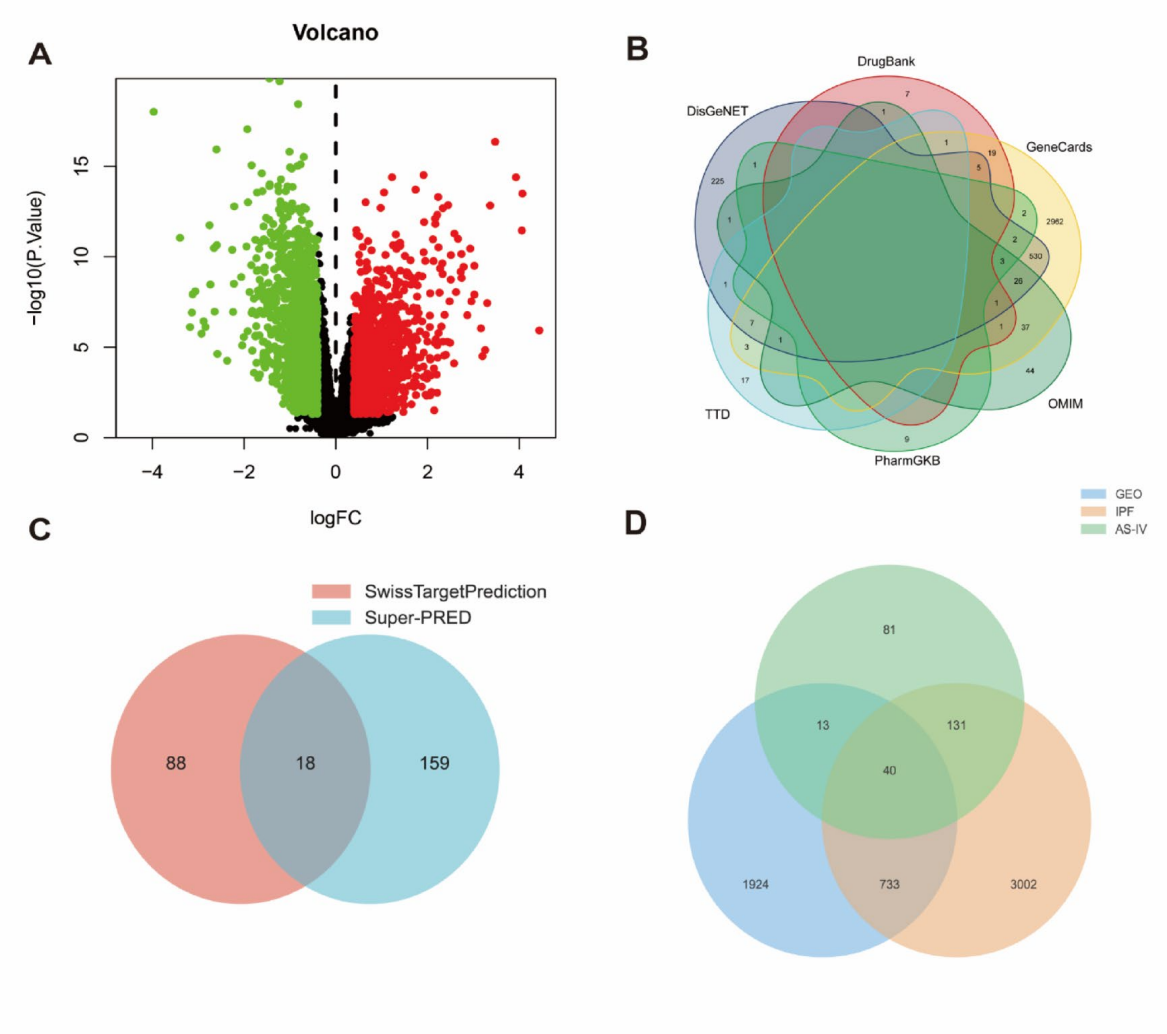
(**A**) Transcriptome volcano plot analysis of clinical samples. Red dots represent upregulated genes, while green dots represent downregulated genes. Black dots indicate genes with no significant differences in expression. (**B**) Network pharmacology analysis of IPF-related genes. (**C**) AS-IV target prediction. (**D**) Venn diagram of overlapping targets across the three datasets.

The GSE53845 microarray dataset, comprising lung tissue samples from 40 patients diagnosed with IPF and 8 healthy individuals, was used to conduct differential gene expression analysis. Utilizing the ‘limma’ package in R, a total of 2710 significantly differentially expressed genes (DEGs) were identified. Among these DEGs, 1332 were upregulated and 1378 were downregulated in IPF lung tissues compared with healthy controls. The distribution of these DEGs, in terms of adjusted *p*-values and log₂ fold changes, is visualized in a volcano plot (Fig. 1A).

#### IPF target genes

To collect IPF-related genes, six databases—GeneCards, OMIM, PharmGKB, TTD, DisGeNET, and DrugBank—were queried using “idiopathic pulmonary fibrosis” as the keyword. A total of 3906 unique disease-related genes were compiled, with the following distributions: GeneCards (3600), OMIM (115), PharmGKB (17), TTD (30), DisGeNET (803), and DrugBank (35) (Fig. 1B).

#### Astragaloside IV targets

AS-IV-related targets were predicted using SUPER-PRED and SwissTargetPrediction databases, yielding 177 and 106 targets, respectively. After eliminating redundancies, 265 unique targets remained (Fig. 1C).

#### Identification of common gene targets

A Venn analysis of the three datasets was performed, resulting in 40 common targets (Fig. 1D).

#### Functional enrichment analysis

**Fig. 2:**
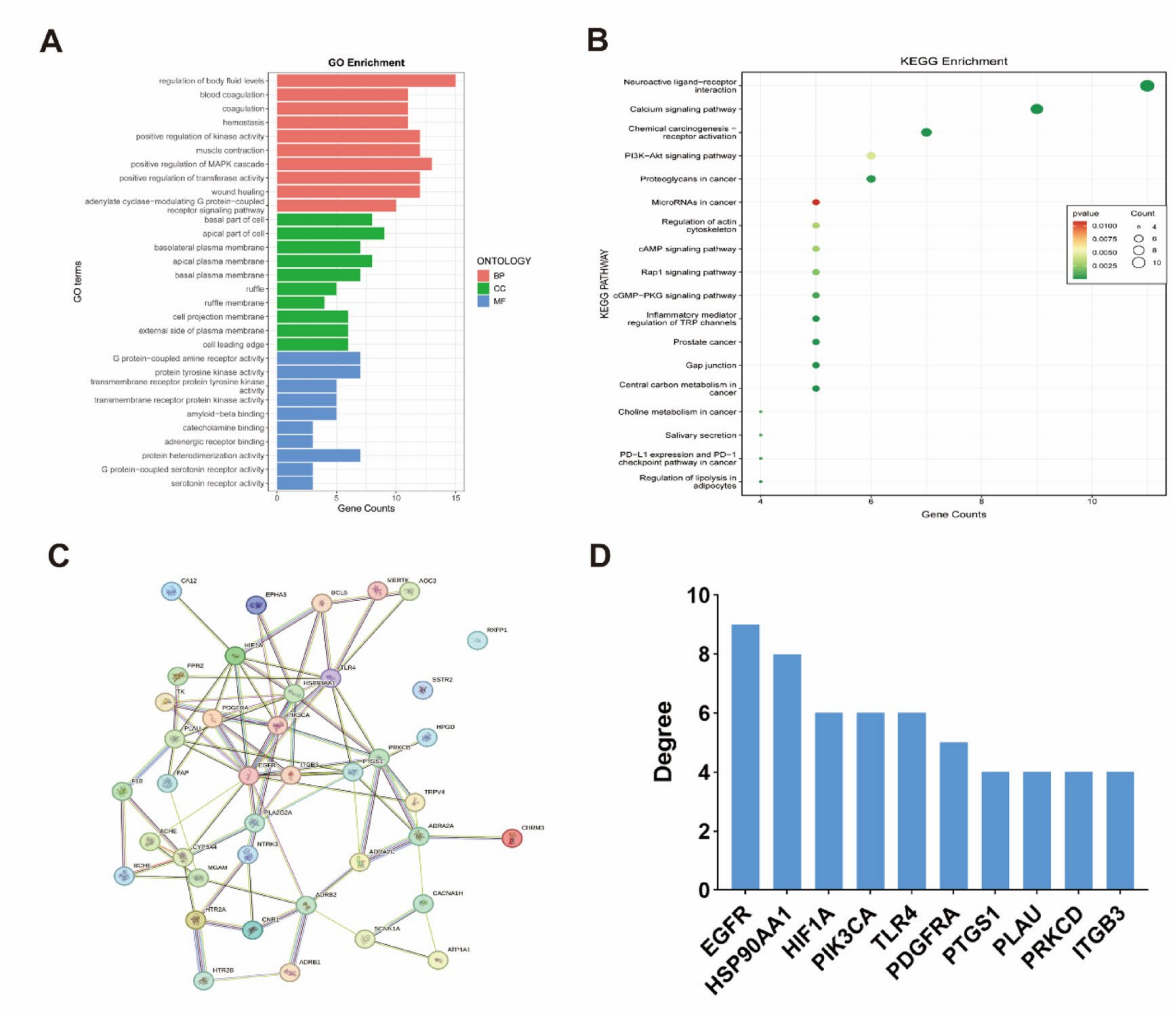
(**A**) Gene Ontology (GO) analysis of common targets (top 10). Red indicates biological processes, green represents cellular components, and green represents molecular functions. (**B**) KEGG pathway enrichment analysis of common targets (top 18). The size of the bubble reflects the pathway count, while the colors indicate the significance of the *p*-value. (**C**) Protein–protein interaction (PPI) network of common targets. (**D**) Degree value ranking of target genes.

Gene Ontology (GO) analysis showed significant enrichment in 508 biological processes (BP), 42 cellular components (CC), and 36 molecular functions (MF) (*p* < 0.05) (Fig. 2A). KEGG pathway enrichment revealed 18 significantly associated pathways, with the PI3K-AKT signaling pathway being strongly implicated in IPF pathogenesis (Fig. 2B).

#### PPI network

To explore interactions among the 40 shared genes, a protein–protein interaction (PPI) network was built using the STRING database (species: Homo sapiens, interaction score > 0.4). The network contained 38 nodes and 84 edges (Fig. 2C). Degree centrality analysis in R identified the top 10 hub genes (Fig. 2D). Notably, PIK3CA—part of the PI3K-AKT pathway—ranked among the top hubs, suggesting it may play a pivotal role in AS-IV’s therapeutic effects against IPF.

#### Molecular docking and molecular dynamics

**Fig. 3:**
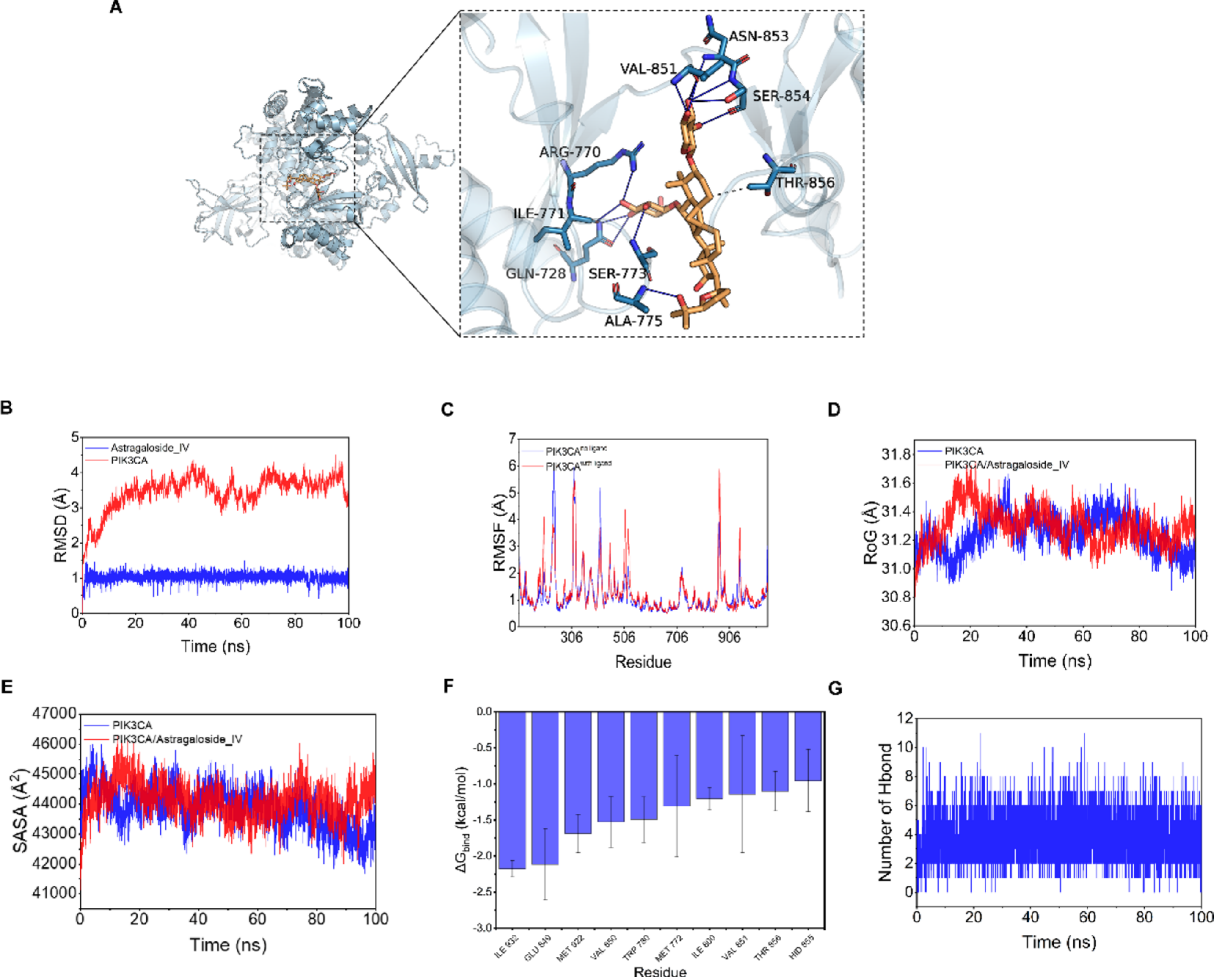
(**A**) Binding mode of AS-IV and PIK3CA during molecular dynamics simulation (MDS). Left: global view; right: local view. Wheat color indicates AS-IV, yellow dashed lines represent hydrogen bonds, and gray dashed lines show hydrophobic interactions. (**B**) Root Mean Square Deviation (RMSD) profile of AS-IV and PIK3CA during MDS. (**C**) Root Mean Square Fluctuation (RMSF) profile of PIK3CA with or without a ligand during MDS. (**D**) Radius of Gyration (RoG) profile of PIK3CA with and without AS-IV during MDS. (**E**) Solvent Accessible Surface Area (SASA) profile of PIK3CA with and without AS-IV during MDS. (**F**) Top 10 amino acids contributing to the binding of small molecules and protein complexes. (**G**) Variations in the number of hydrogen bonds between AS-IV and PIK3CA during MDS.

Given the strong enrichment of the PI3K-AKT pathway, molecular docking was performed to assess the binding of AS-IV to PIK3CA. Hydrogen bonds were observed between AS-IV and residues GLN-728, SER-773, SER-854, VAL-851, ILE-771, ASN-853, ALA-775, and ARG-770, along with hydrophobic interactions with THR-856. The docking score was − 7.3 kcal/mol (Fig. 3A), indicating a favorable binding affinity.

Molecular dynamics simulations validated complex stability. RMSD analysis (Fig. 3B) showed that the protein backbone stabilized after 20 ns, while the ligand (AS-IV) remained stably bound (within 1.5 Å deviation). RMSF analysis (Fig. 3C) showed reduced fluctuations in local regions (e.g., residues 230–239), indicating a stabilizing effect. The radius of gyration (RoG) (Fig. 3D) demonstrated more compact structure upon AS-IV binding. Solvent-accessible surface area (SASA) (Fig. 3E) remained consistent for the complex but decreased for the free protein, implying structural stabilization.

Binding free energy calculated via MM-GBSA was − 44.12 ± 2.53 kcal/mol, mainly driven by van der Waals, electrostatic, and polar solvation contributions (Table 1). Key binding residues included ILE932, GLU849, MET922, VAL850, TRP780, MET772, ILE800, VAL851, THR856, and HID855, with ILE932 and GLU849 contributing more than − 2 kcal/mol (Fig. 3F). Hydrogen bond analysis showed 0–11 bonds during the simulation, with a typical maximum of 4 (Fig. 3G), reinforcing the importance of hydrogen bonding in complex stability.

**Table 1:** Binding free energies and energy components predicted by MM-GBSA method.

System name	AS-IV /PIK3CA
Δ*E*_vdw_	− 55.28 ± 3.11
Δ*E*_elec_	− 30.54 ± 3.44
ΔG_GB_	49.27 ± 2.49
ΔG_SA_	− 7.56 ± 0.34
ΔG_bind_	− 44.12 ± 2.53

ΔE_vdW_: van der Waals energy; ΔE_elec_: electrostatic energy; ΔG_GB_: electrostatic contribution to solvation; ΔG_SA_: non-polar contribution to solvation; ΔG_bind_: binding free energy.

#### AS-IV suppresses phenotypic activation of human lung fibroblasts via PIK3CA in vitro

**Fig. 4:**
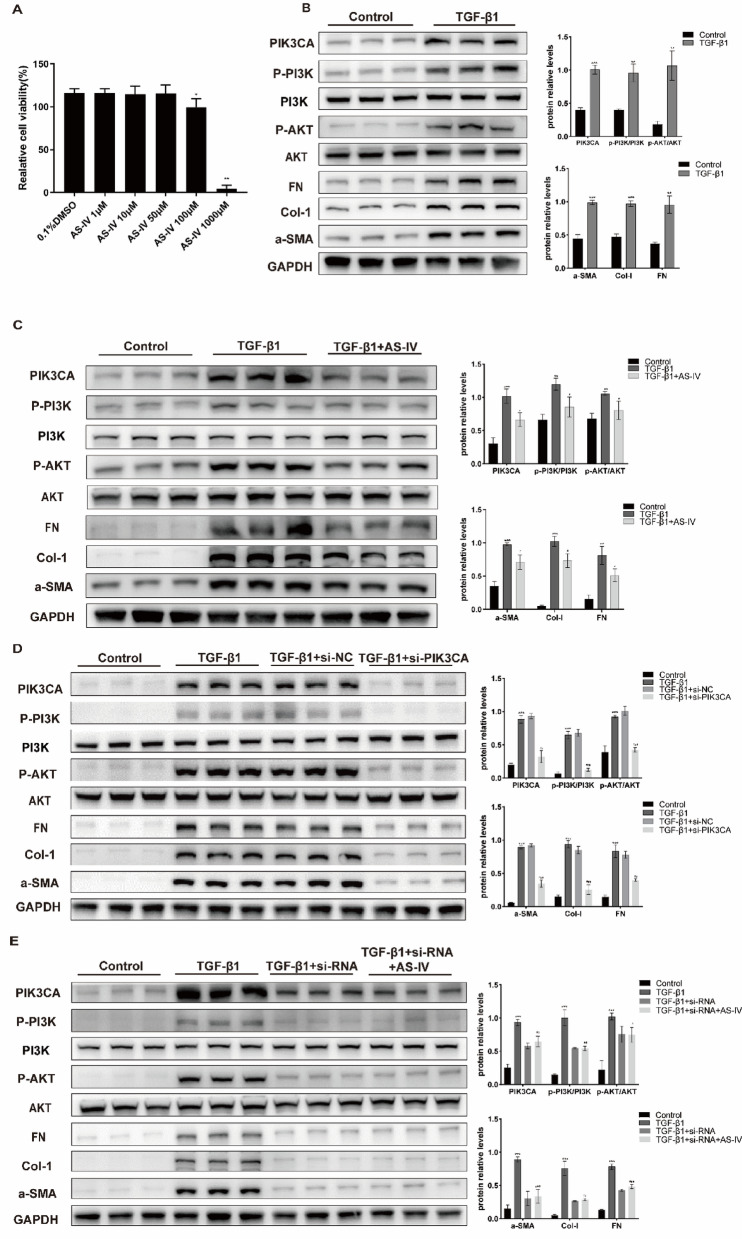
(**A**) Human lung fibroblasts treated with varying concentrations of AS-IV were analyzed using the CCK-8 assay. **p < 0.01, compared to 0.1% DMSO (n = 6). (**B**–**E**) Protein expression levels of PIK3CA, p-PI3K/PI3K, p-AKT/AKT, α-SMA, Col-I, and FN in different intervention groups, followed by semi-quantitative analysis (n ≥ 3). **p < 0.01, compared to control; ***p < 0.001, compared to control; ##*p* < 0.01, compared to TGF-β1; ###*p* < 0.001, compared to TGF-β1.

To investigate whether AS-IV suppresses fibrotic activation in human lung fibroblasts (HLFs), cells were treated with various concentrations of AS-IV for 48 h. CCK-8 assays showed no cytotoxicity at concentrations below 50 μM (Fig. 4A). TGF-β stimulation significantly increased PIK3CA expression and PI3K/AKT pathway activation (evidenced by elevated p-PI3K/PI3K and p-AKT/AKT ratios), along with upregulation of fibrotic markers α-SMA, collagen I, and fibronectin (Fig. 4B). Treatment with 50 μM AS-IV significantly attenuated PIK3CA expression and suppressed PI3K/AKT pathway activation, while also reducing fibrotic marker expression (Fig. 4C). siRNA-mediated knockdown of PIK3CA produced similar inhibitory effects (Fig. 4D). Notably, AS-IV treatment after PIK3CA silencing did not further affect marker levels (Fig. 4E), suggesting AS-IV acts primarily through PIK3CA modulation.

#### AS-IV inhibits pulmonary fibrosis in vivo

**Fig. 5:**
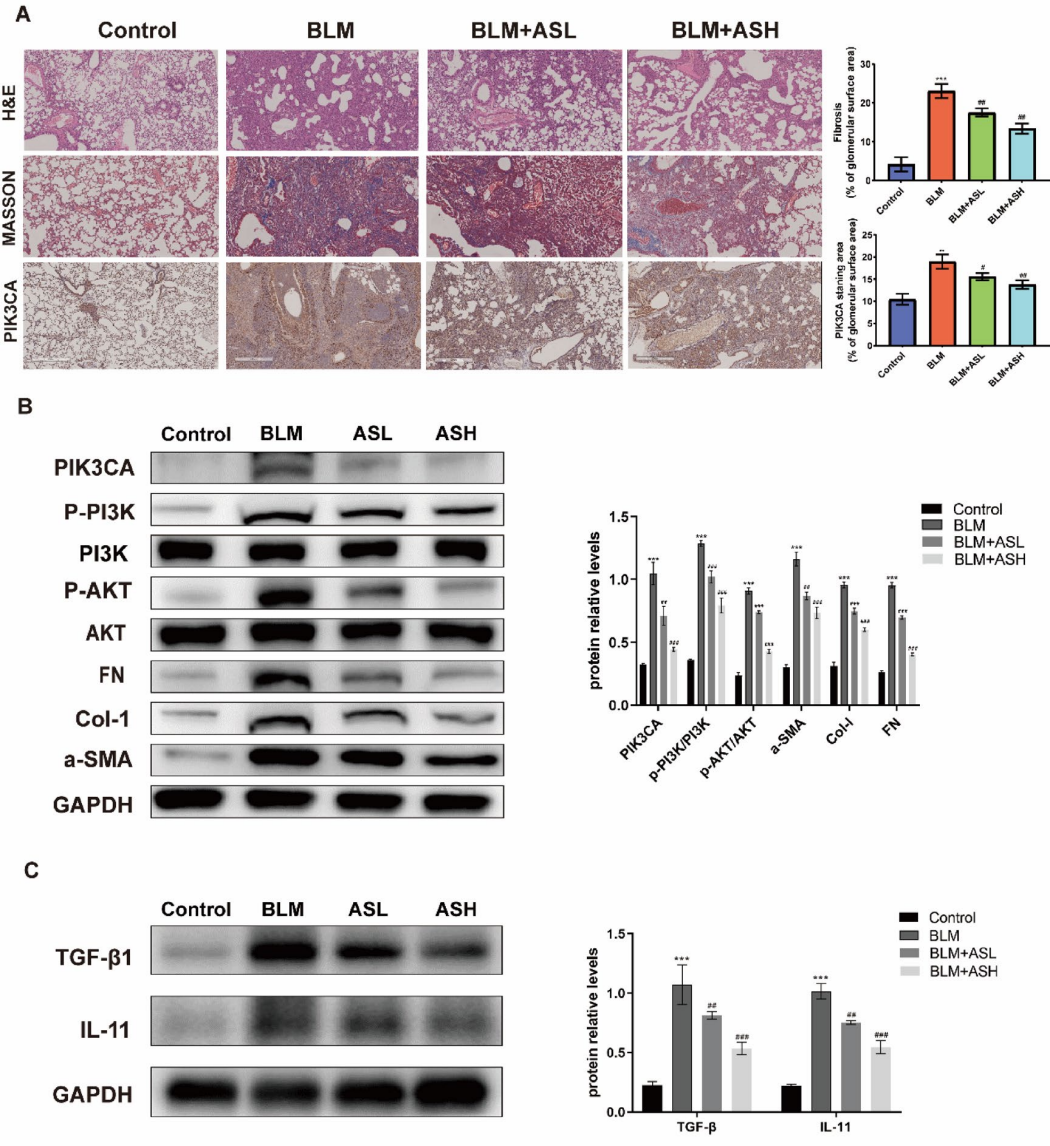
(**A**) Representative images of lung tissue stained with H&E, PAS, Masson, and PIK3CA immunohistochemistry after 14 days of AS-IV treatment (n = 6). Scale bar: 200 μm, original magnification X5. Mean IOD value = total IOD value/total lung tissue area in the same study group. (**B**) Protein expression levels of PIK3CA, p-PI3K/PI3K, p-AKT/AKT, α-SMA, Col-I, and FN in IPF model mice (n = 3). ***p < 0.001, compared to control; ##*p* < 0.01, compared to BLM; ###*p* < 0.001, compared to BLM. (**C**) Protein expression levels of TGFβ1 and IL-11 in IPF model mice (n = 3). ***p < 0.001, compared to control; ##*p* < 0.01, compared to BLM; ###*p* < 0.001, compared to BLM. ASL: BLM + AS-IV low-dose (50 μM), ASH: BLM + AS-IV high-dose (100 μM).

A mouse model of pulmonary fibrosis was established via intratracheal bleomycin (BLM) administration, which typically induces fibrotic features by day 21^24^. H&E staining revealed thickened alveolar septa and fibrotic changes in the BLM group. Masson staining confirmed collagen accumulation (Fig. 5A).

AS-IV was administered orally beginning on day 21 for 14 days. In both low-dose (25 mg/kg) and high-dose (50 mg/kg) AS-IV treatment groups, histological analysis showed attenuated alveolar fibrosis and reduced collagen deposition compared to the BLM group. Immunohistochemical staining showed reduced α-SMA expression, while Western blot analysis demonstrated significant downregulation of PIK3CA, p-PI3K/PI3K, and p-AKT/AKT following AS-IV treatment (*p* < 0.001), particularly in the high-dose group (Fig. 5B). Western blot analysis further confirmed that the expression levels of the inflammatory mediators TGF-β1 and IL-11 were significantly downregulated in the AS-IV-treated groups (*p* < 0.001), supporting their involvement in the PI3K/AKT signaling pathway (Fig. 5C).

## Discussions

In this study, we assessed the therapeutic potential of Astragaloside IV (AS-IV), a primary bioactive compound extracted from *Astragalus membranaceus*, within the framework of idiopathic pulmonary fibrosis (IPF)—a prolonged and degenerative interstitial lung condition characterized by persistent inflammation and abnormal extracellular matrix deposition^25,26^. Despite the availability of antifibrotic agents like pirfenidone and nintedanib, limitations related to efficacy and adverse effects highlight the need for alternative treatments.

Traditional Chinese medicine (TCM) has shown promise in treating IPF, supported by historical evidence and modern pharmacological studies^27,28^.

*Astragalus* has emerged as a particularly noteworthy candidate, with AS-IV identified as a core component capable of modulating fibrosis through multiple signaling pathways^29^, including TGF-β1/PI3K/Akt^30^, RAS/RAF/FoxO^31^, and TGF-β1/Smad2/3^32^. However, the precise mechanisms and key targets of AS-IV in IPF remain incompletely understood.

Network pharmacology provides a comprehensive strategy to decode the multifaceted interactions among drugs, diseases, and targets^33^. In this study, we identified 40 overlapping targets among AS-IV, IPF-related genes, and DEGs from transcriptomic datasets. KEGG enrichment analysis highlighted the PI3K-AKT pathway as a central regulatory axis. Construction of the protein–protein interaction (PPI) network revealed PIK3CA as a central hub, indicating a potentially crucial role in mediating the effects of AS-IV. Subsequent molecular docking and dynamic simulation analyses demonstrated a robust interaction between AS-IV and PIK3CA, with a calculated binding free energy of − 44.12 ± 2.53 kcal/mol. These findings support the hypothesis that AS-IV may exert its antifibrotic effects in part through targeted modulation of PIK3CA.

PIK3CA is a key regulator within the PI3K-AKT signaling cascade and is well-known for its involvement in tumorigenesis^34^, particularly in breast cancer^35^, myocardial injury^36^, and vascular anomalies^37^. In the pathogenesis of pulmonary fibrosis, damaged alveolar epithelial type II (AEC II) cells release substantial quantities of transforming growth factor-beta (TGF-β), a critical mediator initiating the fibrotic cascade^38^. TGF-β induces fibroblast-to-myofibroblast differentiation and activates the TβRI receptor, which subsequently phosphorylates and activates PI3K. PI3K activation results in the production of PIP3, which in turn recruits and activates AKT. Activated AKT mediates phosphorylation of pro-apoptotic proteins, thereby inhibiting apoptosis. This confers resistance to cell death, enabling myofibroblasts to persist in fibrotic lesions, where they continuously secrete excessive extracellular matrix (ECM) components, including collagen and fibronectin, thereby promoting pulmonary scar formation and disrupting the structural integrity of lung. Furthermore, TGF-β and AKT signaling pathways act synergistically to drive epithelial-mesenchymal transition (EMT)^39^. AKT, a central regulator of cellular metabolism, modulates key metabolic pathways, including glycolysis, upon activation by TGF-β, thereby providing essential energy for cell migration and survival^40^. The PI3K/AKT signaling pathway is closely associated with α-SMA overexpression and interacts with the TGF-β pathway^41,42^, contributing to the progression of pulmonary fibrosis through mechanisms such as endoplasmic reticulum stress, autophagy, and EMT^43^. In vivo studies further validated these findings. AS-IV administration reduced α-SMA expression in lung tissues and decreased collagen deposition, as evidenced by Masson staining. In addition, our results showed that AS-IV treatment significantly reduced the expression of inflammatory factors TGF-β and IL-11 closely associated with pulmonary fibrosis, which is consistent with previous studies^44^. These results confirm that AS-IV has notable antifibrotic efficacy in a bleomycin-induced mouse model of IPF.

Molecular dynamics simulations suggest that AS-IV directly binds to PIK3CA, thereby modulating downstream signals and reducing fibroblast phenotypic transformation. In vitro assays confirmed that AS-IV at 50 μM did not induce cytotoxicity but significantly attenuated TGF-β1-induced activation of fibrotic markers. Moreover, siRNA-mediated PIK3CA knockdown diminished the antifibrotic effects of AS-IV, reinforcing that its action is PIK3CA-dependent.

In vivo studies further validated these findings. AS-IV administration reduced α-SMA expression in lung tissues and decreased collagen deposition, as evidenced by Masson staining. These results confirm that AS-IV has notable antifibrotic efficacy in a bleomycin-induced mouse model of IPF.

In summary, AS-IV primarily attenuates the progression of pulmonary fibrosis by modulating the PI3K/Akt signaling cascade via direct engagement with PIK3CA. These results offer a strong conceptual basis for positioning AS-IV as a promising therapeutic candidate for IPF and open new avenues for targeted drug development and clinical investigation.

Nevertheless, this study has certain limitations. First, TCM formulations are inherently complex, and other active components might be generated during preparation. Second, IPF pathogenesis involves multiple interconnected signaling pathways, and the full spectrum of AS-IV’s regulatory effects remains to be clarified. Finally, to comprehensively assess the safety profile of AS-IV, future studies should evaluate organ function across multiple systems in IPF animal models.

### Narrating the future outlook

AS-IV as a naturally-derived antifibrotic compound holds promising prospects for clinical translation. First, clinical trials of AS-IV in IPF patients are needed to systematically evaluate its safety, efficacy, and optimal dosing regimens. Second, further optimization of AS-IV pharmaceutical formulations and delivery routes, such as developing targeted pulmonary inhalation preparations, could enhance drug concentrations at disease sites while reducing systemic adverse effects. Third, detailed elucidation of the precise molecular mechanisms underlying AS-IV binding to PIK3CA will provide guidance for structure-based drug design. Fourth, exploration of combination therapy strategies with Pirfenidone or Nintedanib may enhance antifibrotic effects through multi-target synergistic actions. Finally, development of companion diagnostic technologies to identify IPF patients most likely to benefit from AS-IV treatment through detection of PIK3CA expression levels or PI3K pathway activation status will enable truly personalized precision therapy. These research directions will facilitate the successful translation of AS-IV from laboratory discovery to clinical application.

## Data availabilty

The data generated in the present study may be requested from the corresponding author.

## Supplementary Information

Below is the link to the electronic supplementary material.

Supplementary Material 1
