# Supplementary figures and images for: Integrative analysis of the therapeutic mechanisms of Astragaloside IV in idiopathic pulmonary fibrosis via network pharmacology and molecular validation
Source: Sci Rep. 2025 Nov 12;15:39624. doi: 10.1038/s41598-025-23354-8 (PMC12612232; doi:10.1038/s41598-025-23354-8)

Fig4B

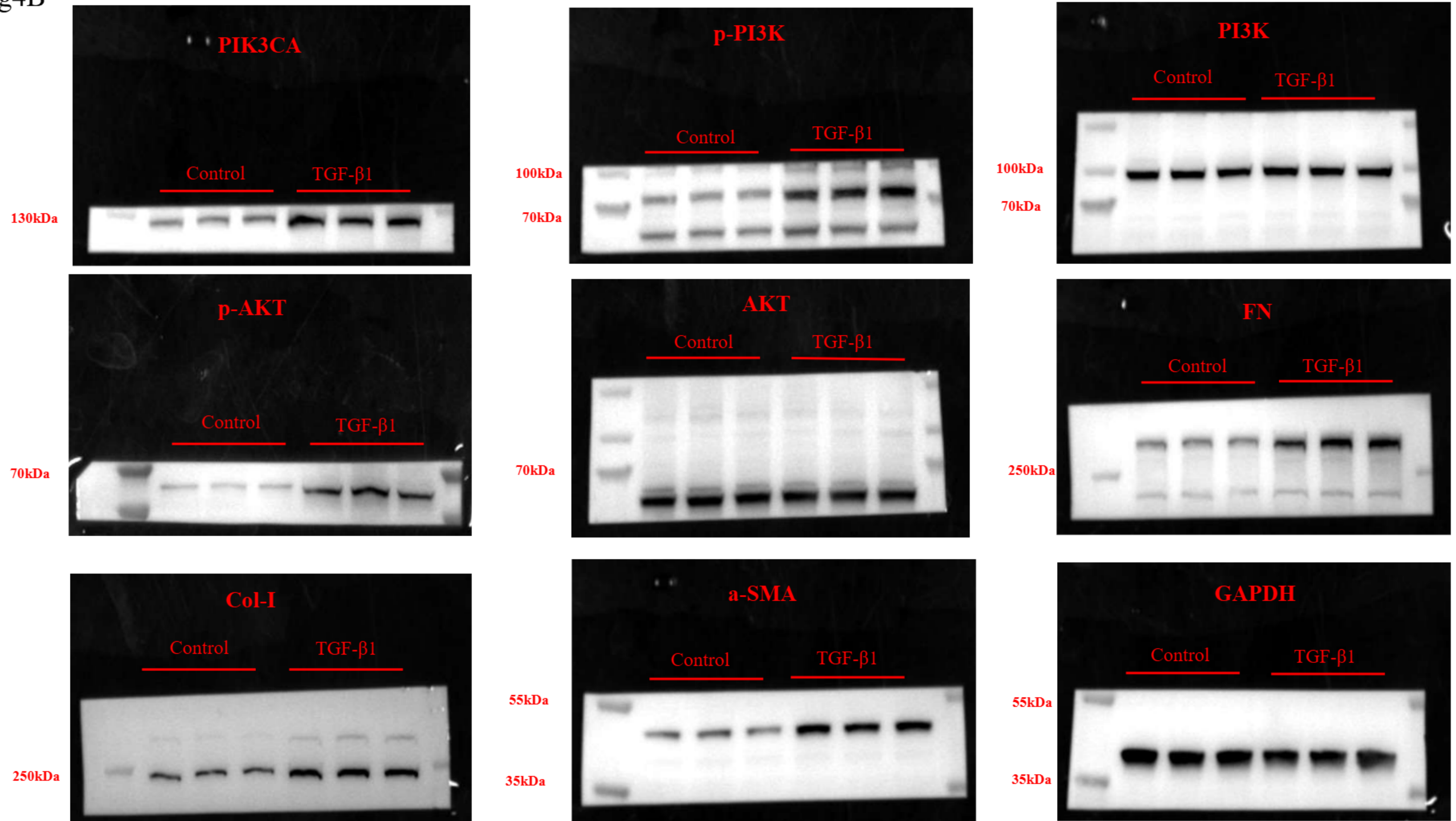

Fig4C

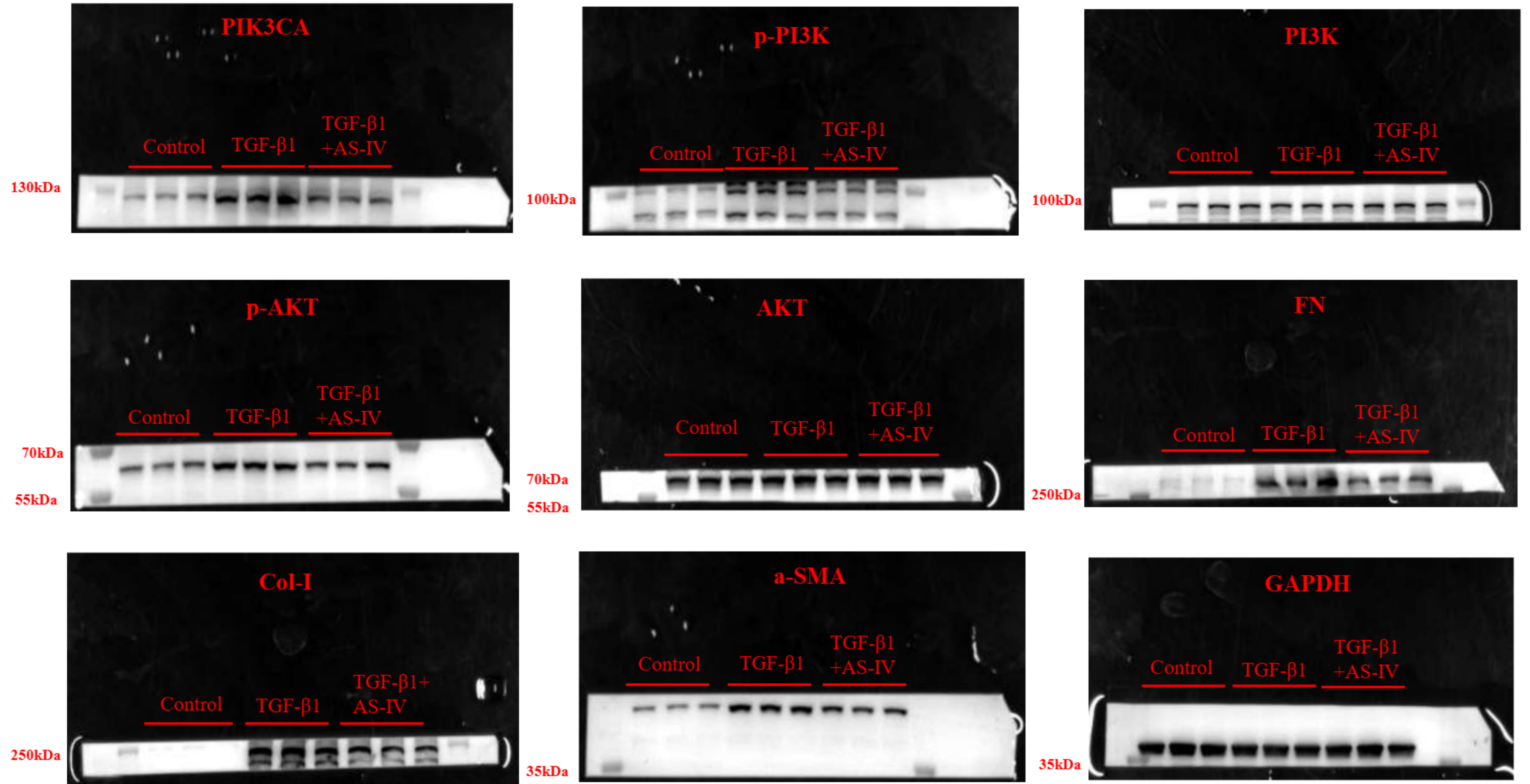

Fig4D

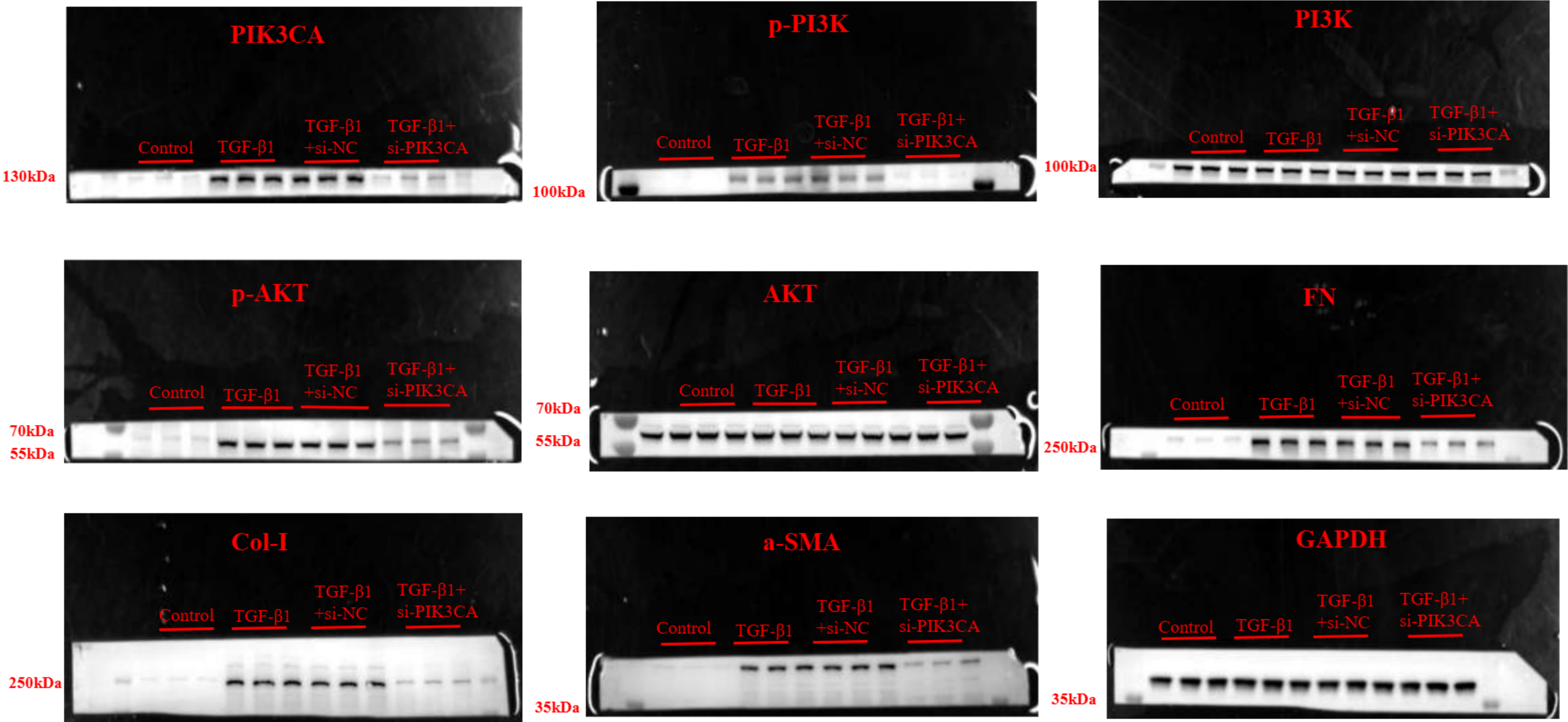

Fig4E

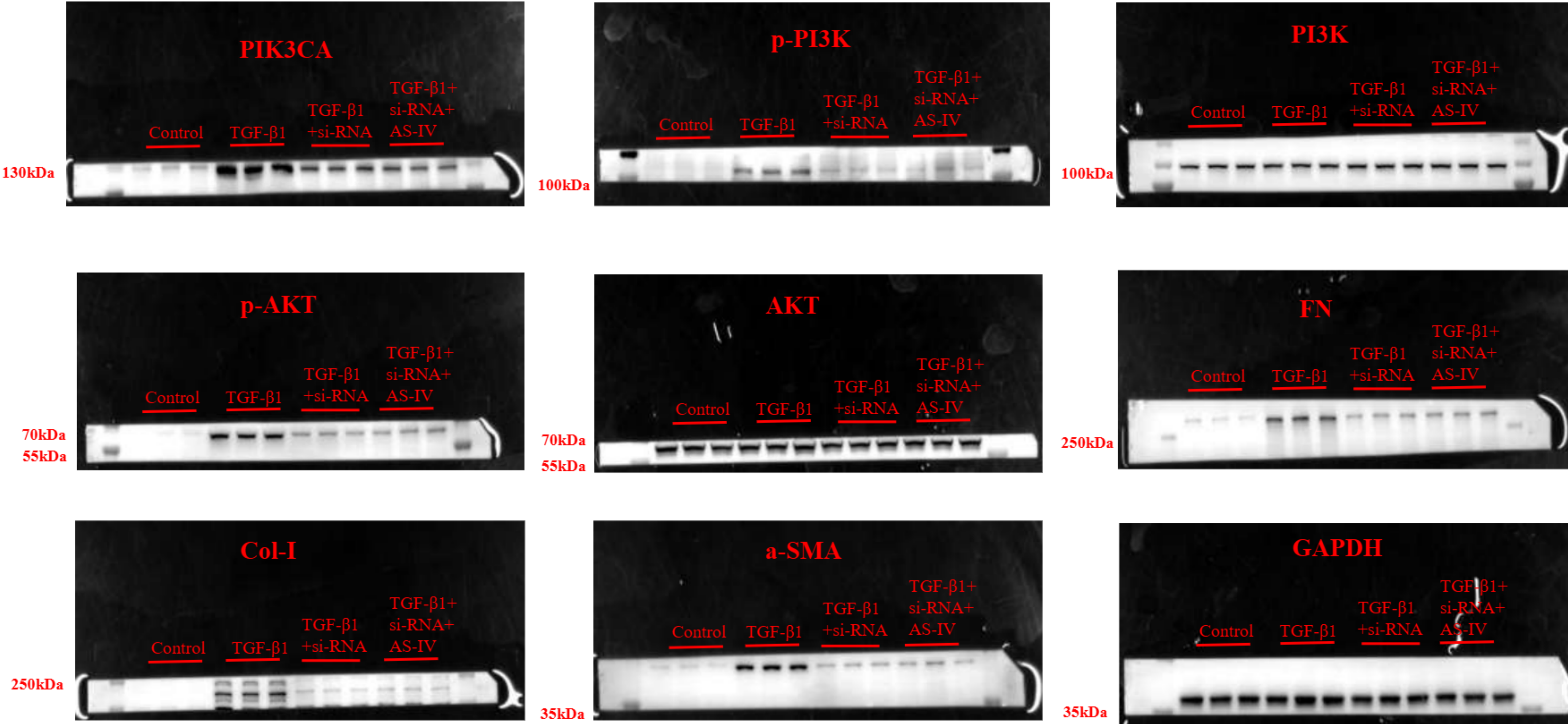

Fig5B

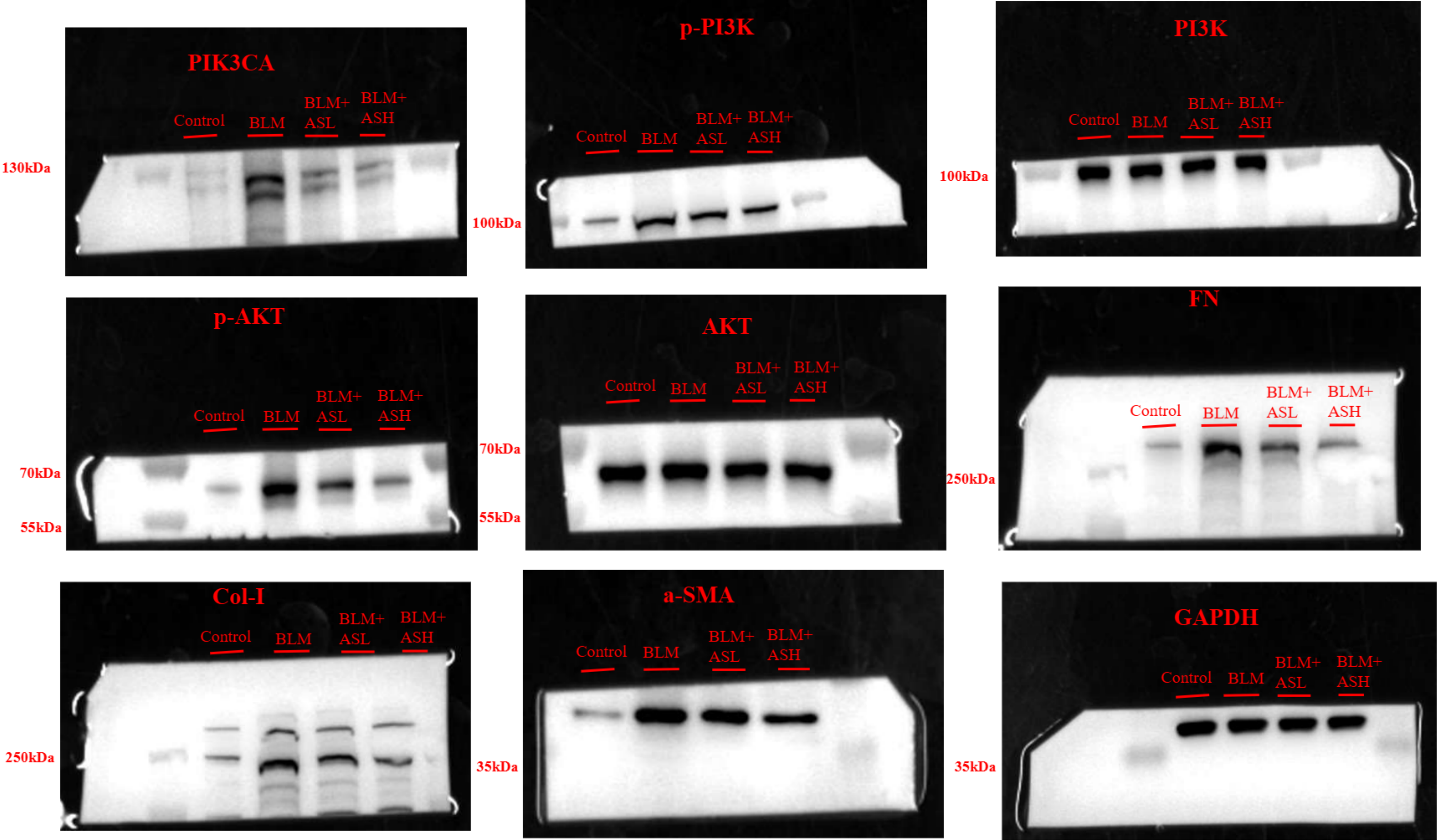

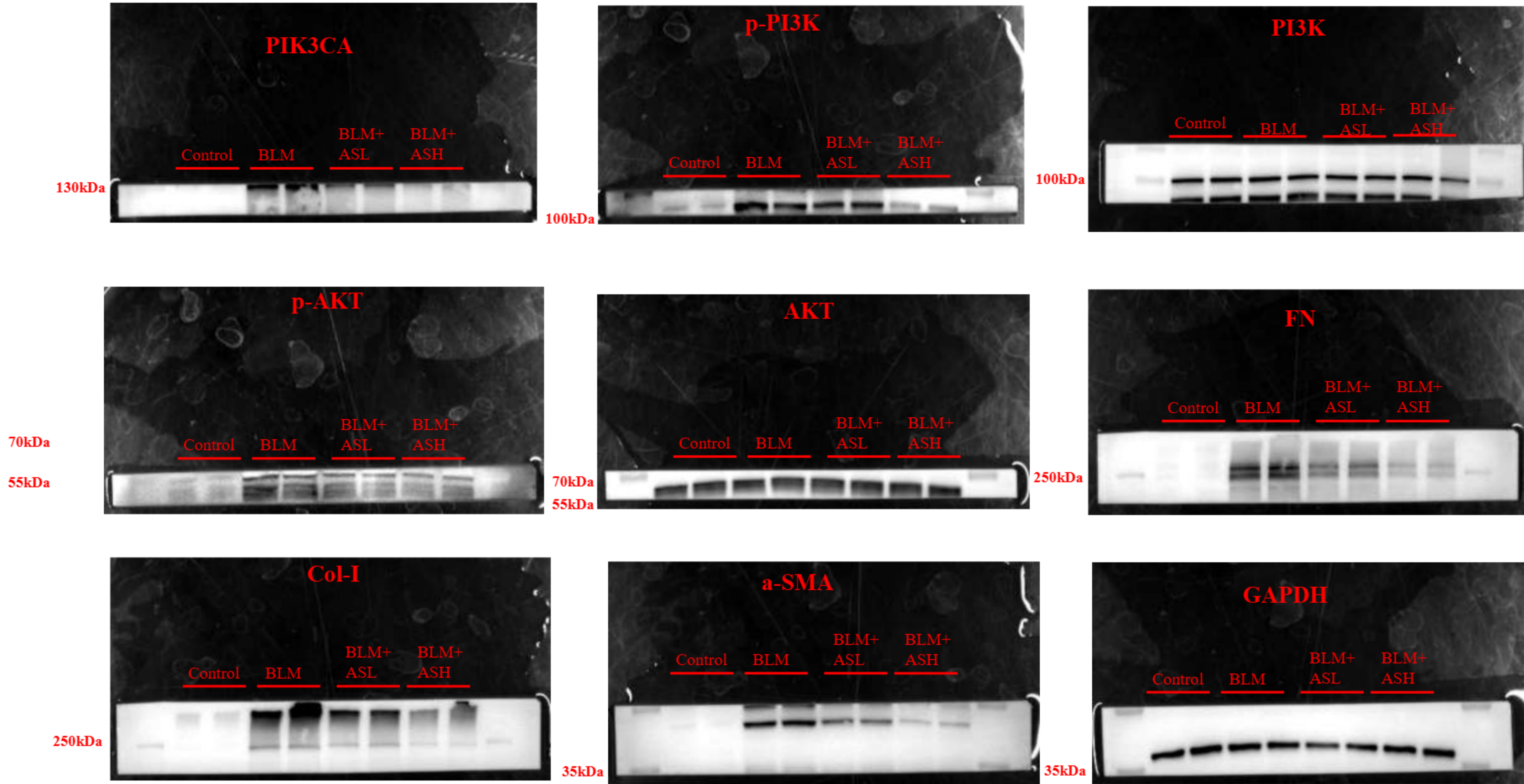

Fig5C

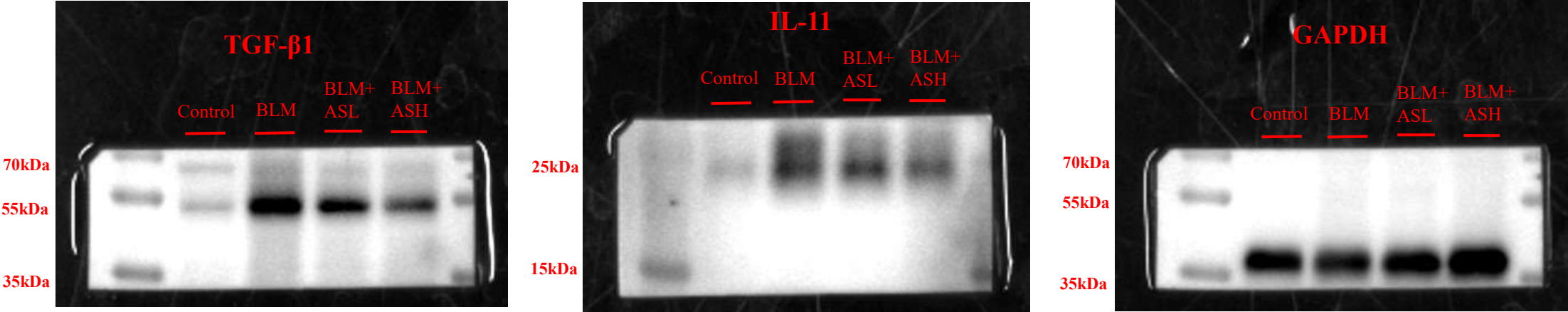

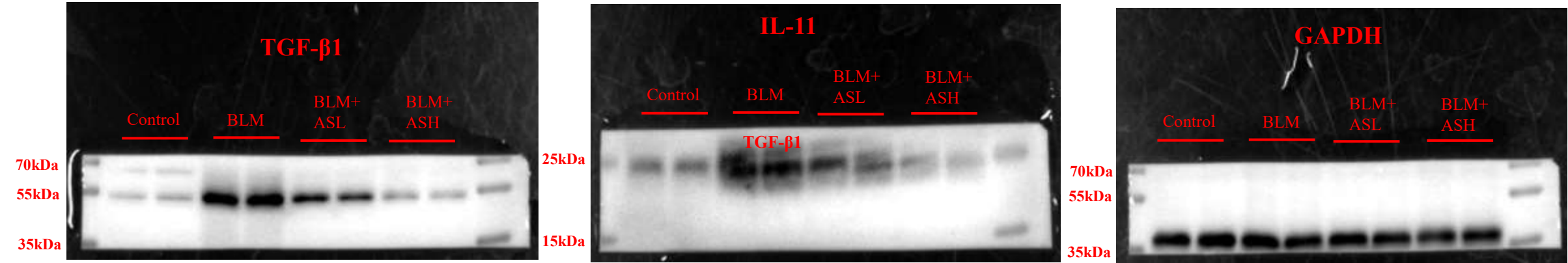

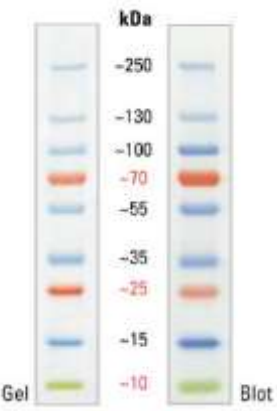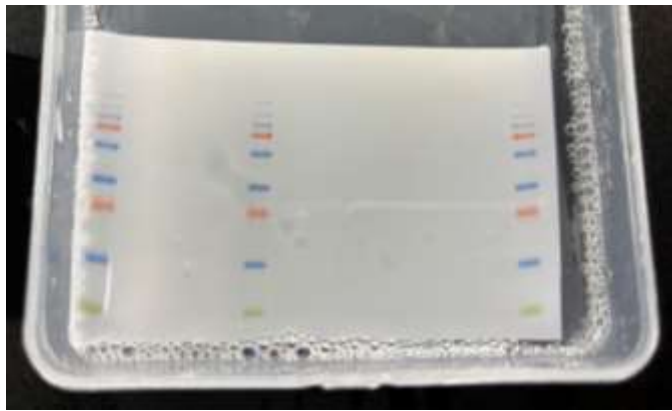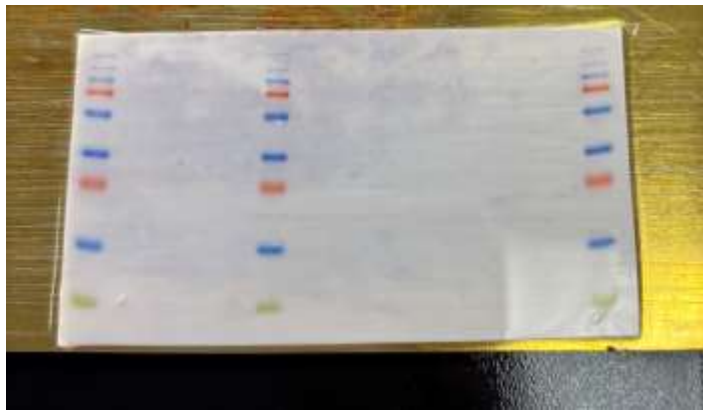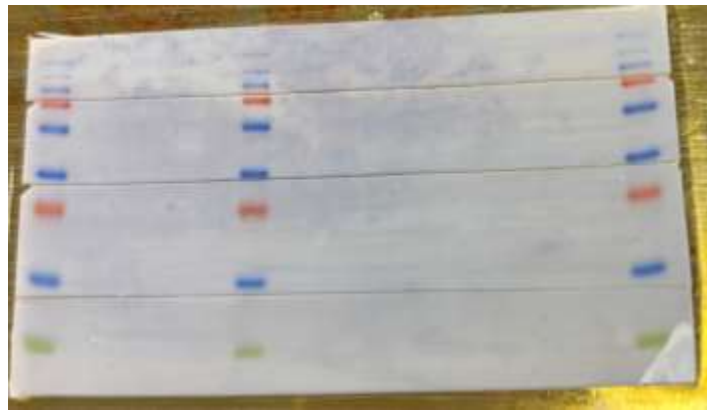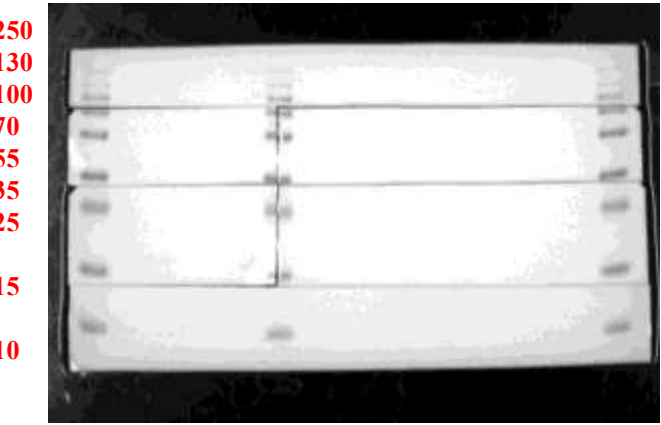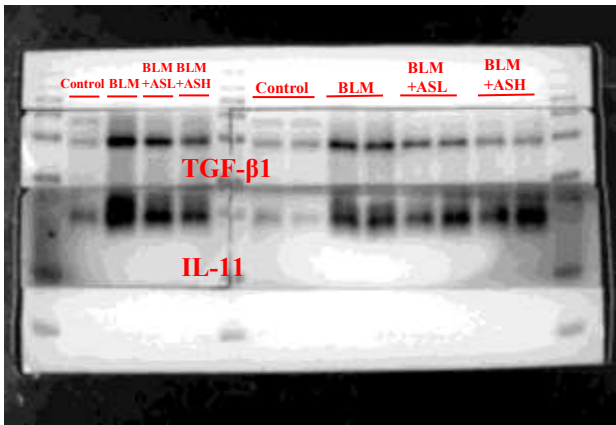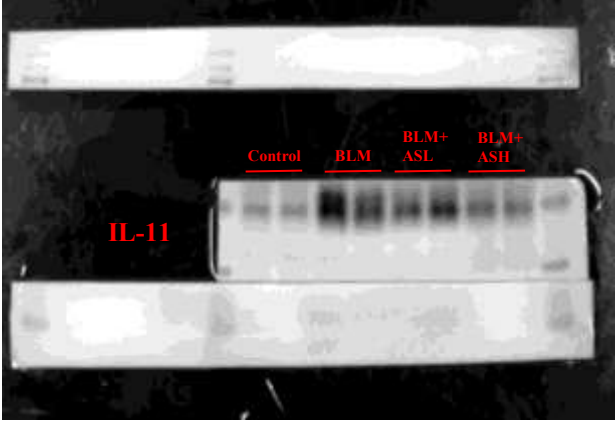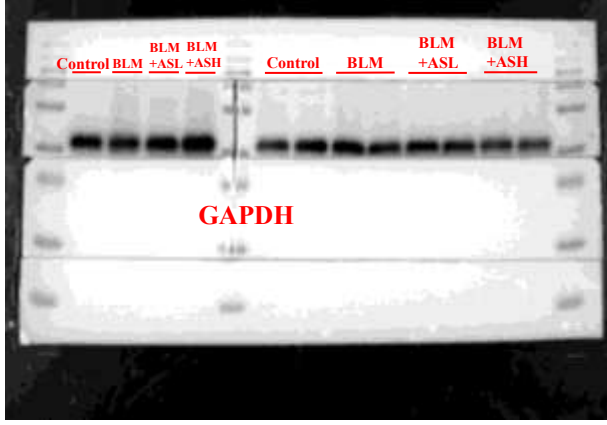

Supplement: Supplementary file 1 — Supplementary Material 1 [file 41598_2025_23354_MOESM1_ESM.pdf]
